# Catecholamine responses to virtual combat: implications for post-traumatic stress and dimensions of functioning

**DOI:** 10.3389/fpsyg.2015.00256

**Published:** 2015-03-17

**Authors:** Krista B. Highland, Michelle E. Costanzo, Tanja Jovanovic, Seth D. Norrholm, Rochelle B. Ndiongue, Brian J. Reinhardt, Barbara Rothbaum, Albert A. Rizzo, Michael J. Roy

**Affiliations:** ^1^Center for Neuroscience and Regenerative Medicine, Uniformed Services University of the Health Sciences, Bethesda, MDUSA; ^2^Department of Medicine, Uniformed Services University of the Health Sciences, Bethesda, MDUSA; ^3^ Henry Jackson Foundation, Bethesda, MDUSA; ^4^Department of Psychiatry and Behavioral Sciences, Emory University School of Medicine, Atlanta, GAUSA; ^5^Atlanta Veterans’ Affairs Medical Center, Decatur, GAUSA; ^6^Walter Reed National Military Medical Center, National Intrepid Center of Excellence, Bethesda, MDUSA; ^7^Department of Research Programs, Walter Reed National Military Medical Center, Bethesda, MDUSA; ^8^Exploratory Center for the Interdisciplinary Study of Neuroplasticity and Stroke Rehabilitation, University of Southern California, Los Angeles, CA, USA

**Keywords:** norepinephrine, posttraumatic stress, PTSD, functional status, virtual reality

## Abstract

Posttraumatic stress disorder (PTSD) symptoms can result in functional impairment among service members (SMs), even in those without a clinical diagnosis. The variability in outcomes may be related to underlying catecholamine mechanisms. Individuals with PTSD tend to have elevated basal catecholamine levels, though less is known regarding catecholamine responses to trauma-related stimuli. We assessed whether catecholamine responses to a virtual combat environment impact the relationship between PTSD symptom clusters and elements of functioning. Eighty-seven clinically healthy SMs, within 2 months after deployment to Iraq or Afghanistan, completed self-report measures, viewed virtual-reality (VR) combat sequences, and had sequential blood draws. Norepinephrine responses to VR combat exposure moderated the relationship between avoidance symptoms and scales of functioning including physical functioning, physical-role functioning, and vitality. Among those with high levels of avoidance, norepinephrine change was inversely associated with functional status, whereas a positive correlation was observed for those with low levels of avoidance. Our findings represent a novel use of a virtual environment to display combat-related stimuli to returning SMs to elucidate mind-body connections inherent in their responses. The insight gained improves our understanding of post-deployment symptoms and quality of life in SMs and may facilitate enhancements in treatment. Further research is needed to validate these findings in other populations and to define the implications for treatment effectiveness.

## Introduction

Symptoms of posttraumatic stress disorder (PTSD) are common in the aftermath of war, and the recent wars in Iraq and Afghanistan are no exception (Veterans Health Administration, 2014). Exposure to traumatic experiences such as being attacked, ambushed, or in an accident, or witnessing physical devastation during these campaigns has been associated with PTSD, depressive symptoms, hazardous drinking, and relationship stress, which persist for at least 9 months after returning home (Cigrang et al., 2014). PTSD symptoms resulting from combat-related events can impair quality of life and functional status (e.g., degree of disability across domains of health and behavior) among service members (SMs; Schnurr et al., 2009), including those who are subthreshold, or lacking a sufficient number or distribution of symptoms to meet full criteria for PTSD (Magruder et al., 2004; Grubaugh et al., 2005; Cukor et al., 2010). PTSD symptom clusters include re-experiencing (e.g., intrusive memories and bad dreams), avoidance (e.g., avoidance of trauma-related thoughts, feelings, activities, as well as a loss of social connection, and interest in activities), and hyperarousal (e.g., strong startle response, sleep problems, angry outbursts, hypervigilance), with dysphoric mood recently added. While PTSD is associated with poorer functional outcomes overall, this relationship may vary according to particular symptom clusters and domains of functional status. The intent of this report is to explore the relationship between PTSD symptom clusters and functional impairment through an assessment of blood catecholamine responses to a combat-related virtual environment in a sample of U.S. military SMs recently returned from deployment.

Posttraumatic stress disorder symptoms are multifaceted and the number and intensity of symptoms can vary considerably between individuals resulting in variable patterns of functional impairment (Norrholm and Jovanovic, 2010). Previous studies suggest the importance of examining PTSD symptom clusters separately, rather than one amalgamated score. In studies of veterans of the Iraq and Afghanistan wars, dysphoria has been linked to a multitude of poor outcomes including mental health function, problematic alcohol use, and greater psychosocial, and work difficulties (Pietrzak et al., 2010), as well as greater propensity to seek mental health care (Blais et al., 2014). Re-experiencing symptoms have been positively associated with alcohol use problems (Pietrzak et al., 2010), healthcare utilization (Blais et al., 2014), reduced health functioning, and elevated bodily pain (Asnaani et al., 2014); while avoidance was linked with greater psychosocial difficulties (Pietrzak et al., 2010) and lower healthcare utilization (Blais et al., 2014). Hyperarousal symptoms have been independently associated with lower vitality and emotional functioning (Asnaani et al., 2014). Though PTSD symptom clusters have sometimes been associated with functional deficits, they are not invariably associated with impaired function, and psychophysiological factors may represent important moderators.

The sympathetic nervous system stimulates the release of the catecholamines epinephrine, norepinephrine, and dopamine to mediate adaptive responses to acute stressors (Southwick et al., 2002; Cahill and Alkire, 2003), but they are also linked with long-term memory of events that induce strong emotions such as fear (Soeter and Kindt, 2011). Though adaptive for acute stress, chronic stress, and associated repetitive catecholamine-system activation leads to damaging biopsychosocial outcomes (Mead et al., 2010). In a community sample, individuals with PTSD had higher 24-h levels of catecholamines compared to both those without trauma exposure, as well as those exposed to trauma who did not develop PTSD (Young and Breslau, 2004). In fact, those with trauma exposure who did not develop PTSD actually had lower catecholamine levels than those without trauma exposure (Young and Breslau, 2004), indicating a potential mechanism for resilience. Though catecholamine levels are related to PTSD symptomatology, research on catecholamine responses to acute combat-related cues among post-deployment SMs is limited. In a small study of veterans with and without PTSD, those with PTSD had exaggerated epinephrine and norepinephrine responses to combat-sounds than those without PTSD (Liberzon et al., 1999). Recent technological advances make it possible to present salient combat-related stimuli and monitor individualized psychophysiological reactions, providing the potential to elucidate the complexities of trauma-related symptomology and functioning outcomes.

Virtual reality exposure therapy (VRET) has shown efficacy in the treatment of combat-related PTSD (Roy et al., 2010; McLay et al., 2011; Reger et al., 2011; Goncalves et al., 2012; McLay et al., 2014). A study conducted in U.S. veterans with recent-onset combat-related PTSD found both that virtual reality (VR) was acceptable to them, and that the psychological, physiological, and behavioral responses it elicited were similar to those they had experienced during deployment (Kramer et al., 2013). However, there is relatively little in the medical literature regarding the use of VR in PTSD assessment. Recently, one study examined responses to neutral computer-generated characters using head-mounted display VR in participants who had experienced a physical assault 4 weeks prior, which found that reactions to the neutral stimuli predicted paranoia and PTSD 6 months later (Freeman et al., 2014). We previously documented a relationship between physiologic responses to combat-related VR scenarios and PTSD symptoms (Roy et al., 2013; Costanzo et al., 2014), but we believe that incorporation of catecholamine measurements in the analysis can further improve our understanding of the impact of exposure to virtual combat sequences. We therefore examine catecholamine levels (epinephrine, norepinephrine) before and after exposure to virtual combat sequences in relation to PTSD symptom clusters (e.g., re-experiencing, avoidance, hyperarousal) and multiple functional domains (e.g., mental health, physical health, vitality). We hypothesize that catecholamine responses to virtual combat sequences will positively correlate with PTSD symptom clusters and that each will be inversely correlated with functional status.

## Materials and Method

### Participants and Procedures

The present study was approved by the institutional review boards at the Walter Reed National Military Medical Center, Uniformed Services University of the Health Sciences, and the National Institutes of Health, and adhered to the Declaration of Helsinki. Clinically healthy SMs within 2 months of return from deployment to either Iraq or Afghanistan first completed self-report questionnaires, then viewed VR combat sequences, and completed baseline and post-VR blood draws for catecholamines. Participants viewed 3 sequential 2-min Virtual Iraq prescripted sequences (Rizzo et al., 2010) on a computer screen, which were separated by 30-s presentations of a blue square; the sequences, respectively, depicted a first person view at the gunner position of a Humvee, within the cabin of a Humvee, and walking through the streets of a middle eastern city. The scenarios included smoke, gunfire, explosions, and overhead aircraft.

Blood was drawn from each participant prior to 9:00 AM (baseline), as well as immediately after exposure to the three virtual combat sequences. Plasma samples were isolated from whole blood in sodium heparin preserved collection tubes with centrifugation for 10 min at room temperature. Samples were stored at -70°C, and all samples were then run in a single batch. Following manufacturer’s instructions, a commercially available enzyme immunoassay was used to measure plasma catecholamine levels, including epinephrine and norepinephrine. First, catecholamines were extracted with a cis-diol-affinity specific gel, followed by acetylation and enzymatic conversion. Then, the separate microtiter plates bound with specific catecholamine antigen were processed with a competitive enzyme linked immunosorbent assays. Standards, controls, samples, and their respective antiserum were incubated for 20 h, followed by washing with the automated plate washer to remove free antigen-antiserum complexes. An anti-rabbit IgG-peroxidase conjugate using 3.3^′^, 5.5^′^ Tetramethylbenzidine (TMB) as the substrate detected antibody, and a plate reader then detected absorbance at 450 nm. Next, sample and standard curve analysis was calculated. A non-linear regression, 4-parameter logistic curve fitting method calculated the standard curve and sample concentrations (Molecular Devices Corporation, 2012). The equation used to generate this curve fit is: y = ((A - D) / (1 + (× /C) B)) + D.

### Measures

Posttraumatic stress disorder symptom clusters were assessed with the 17-item PTSD Checklist Military Version (PCL-M), for which the total score may range from 17 to 85, with the various items measuring symptoms within each of the 3 clusters. The PCL-M compares favorably with the gold-standard Clinician Administered PTSD Scale (Forbes et al., 2001). Functional status was assessed using the widely used and well-validated 36-item Short Form Health Survey (SF-36; Ware and Sherbourne, 1992). Epinephrine and norepinephrine values at baseline were subtracted from values after VR so that “catecholamine responses” represent the difference in values between the two time points.

### Analyses

Demographic characteristics and biopsychosocial factors were assessed by univariate analysis to obtain frequencies for categorical variables and means and standard deviations for continuous variables. We then examined the relationships between PTSD symptom clusters, catecholamine responses, and functional status subscales in a multivariate fashion with a series of multiple linear regressions. A PCL-M cluster (centered) was placed in Block 1 and a catecholamine response value (centered) was placed in Block 2. To examine moderating effects, the respective PCL-M cluster X catecholamine response interaction term was placed in Block 3. To control for the risk of false positive findings, we utilized an online False Discovery Rate calculator (http://sdmproject.com/utilities). All statistical analyses were completed using IBM SPSS (version 22).

## Results

### Univariate and Bivariate Findings

**FIGURE 1:**
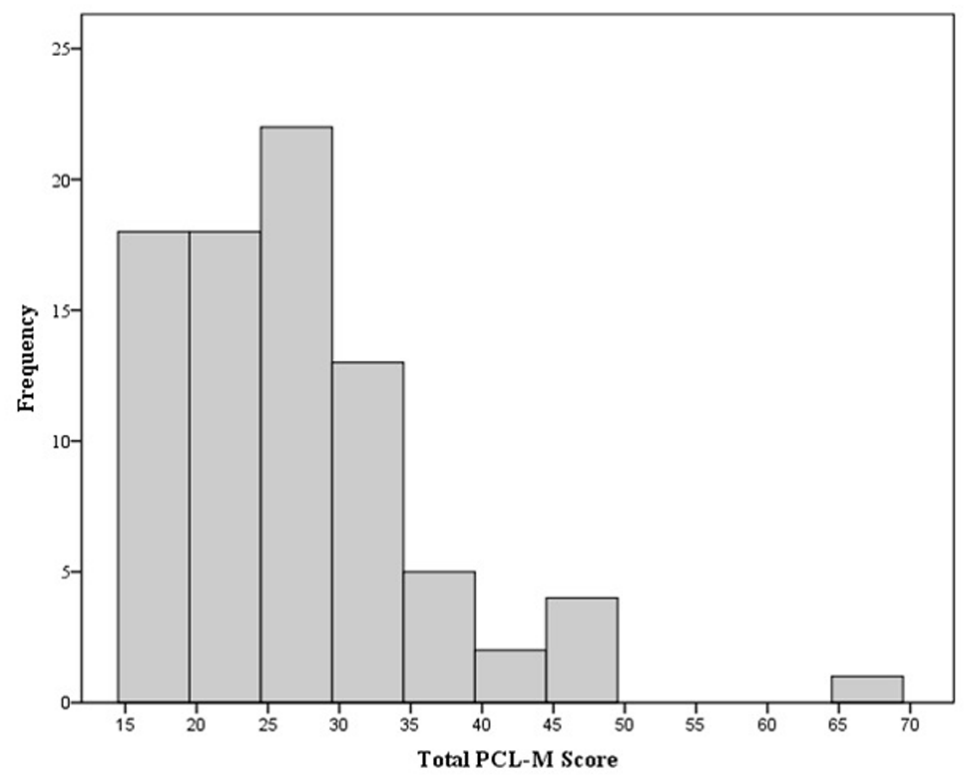
**Frequency of PCL-M total scores**.

Study participants (*N* =87) were predominantly male (80%) and self-identified as white (73%); all were either active or reserve component SMs representing multiple military service branches. On average, participants were 30.0 (SD = 8.0) years of age, had 9.4 (SD = 5.9) years of service, and had been deployed 1.7 (SD = 0.95) times. The average PCL-M total score was 26.93 (SD = 8.74); one participant had a score > 50 and was excluded from the physiological analysis, 50 is a widely used cut-off for identifying a likely diagnosis, while another two had a PCL-M score within 5% of that cut-off. The frequency of PCL-M scores are shown in a histogram (**Figure 1**). On average, epinephrine levels increased by 2.2% after VR, whereas norepinephrine levels decreased by 11.2%. Means and SD are shown in **Table 1**.

**Table 1:** Means and SD for catecholamine, trauma symptoms, and functioning scores.

Variable	Mean (SD)
Baseline epinephrine	51.30 (25.57)
Epinephrine response	1.20 (36.13)
Baseline norepinephrine	274.77 (173.50)
Norepinephrine response	-38.50 (200.30)
PCL-M total	26.93 (8.74)
PCL re-experiencing	7.22 (2.47)
PCL avoidance	8.92 (2.30)
PCL hyperarousal	8.76 (3.52)
Bodily pain	77.73 (16.93)
General health	82.32 (13.83)
Mental health	79.71 (11.38)
Physical functioning	94.69 (11.56)
Emotional role functioning	85.37 (27.27)
Physical role functioning	92.68 (16.89)
Social functioning	83.02 (17.17)
Vitality	63.90 (15.34)

*PCL-M = Posttraumatic stress disorder (PTSD) checklist – military.*

There were several significant correlations among predictor and outcome variables. When looking at the relationship between PCL-M total score and outcomes, we found that higher PCL-M scores were associated with more bodily pain (*r* = -0.37, *p* < 0.01), and poorer general health (*r* = -0.24, *p* < 0.05), mental health (*r* = -0.38, *p* < 0.01), emotional role functioning (*r* = -0.24, *p* < 0.05), social functioning (*r*= -0.57, *p*< 0.01), and vitality (*r* = -0.40, *p* < 0.01). When examining these correlations on a symptom cluster level, higher levels of hyperarousal was related to more bodily pain (*r* = 0.26, *p* < 0.05) and poorer general health (*r*= -0.35, *p* < 0.01). There were no *direct* linear effects observed between catecholamine responses and functioning status subscales.

### Linear Regression

**FIGURE 2:**
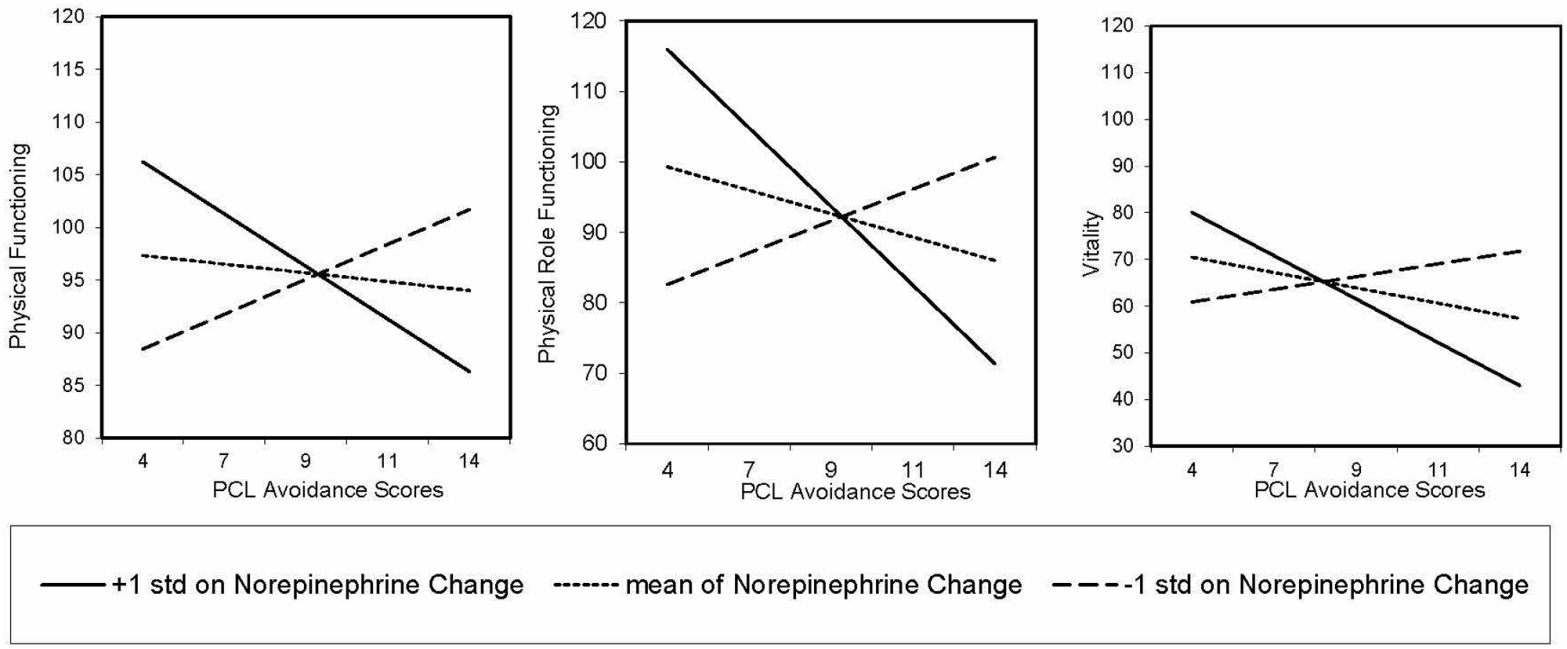
**Pattern of relationship between PCL-M avoidance and physical functioning, physical role functioning, and vitality as moderated by norepinephrine response from baseline to post-virtual reality**.

A series of simple and multiple linear regressions examined the relationship between PCL-M symptom clusters and catecholamine responses with functional status subscales. We used a false discovery rate correction for multiple comparisons (*q*-values) to determine significance (i.e., *p* < 0.05 and *q*< 0.10). First, we assessed whether PCL-M symptom clusters and catecholamine responses were independently associated with functional status subscales using several simple linear regressions. We found a significant main effect for hyperarousal scores on general health (β = -0.35, *p* = 0.001, *q* = 0.01), such that as hyperarousal increased, reported general health decreased. No other main effects were significant. Next, we examined the independent and interactive effects of PCL-M symptom clusters and catecholamine responses on functional status subscales using several multiple linear regressions. Consistent with our hypothesis, norepinephrine responses to VR combat sequences significantly moderated the relationship between avoidance and functional status subscales, including physical functioning (β = 0.53, *p* < 0.001, *q* = 0.001), physical role functioning (β = 0.36, *p* = 0.002, *q* = 0.02), and vitality (β = 0.36, *p* = 0.002,* q =*0.02; **Figure 2**). For individuals with lower avoidance symptoms, increased norepinephrine responses were associated with increased functional status subscale scores, while participants with higher avoidance symptoms, increased norepinephrine responses were associated with decreased functional status subscale scores.

## Discussion

Norepinephrine release in response to virtual combat sequences modulates the relationship between the avoidance symptoms of PTSD and multiple domains of functional status in a population of recently deployed U.S. military SMs. For individuals with lower levels of avoidance symptoms, a significant catecholamine response was indicative of better functional status. However, for SMs reporting more severe avoidance symptoms, an amplified catecholamine response was associated with poorer functional status across multiple domains. From the perspective of emotional processing theory, avoidance reduces opportunities to dispel trauma-related, maladaptive beliefs, enabling the persistence of hyperarousal and re-experiencing symptoms that may have been normal initial reactions (Rauch and Foa, 2006). Participants having both higher avoidance symptoms and high catecholamine responses to trauma-related stimuli may be disengaging from day-to-day experiences, which may in turn adversely impact their physical function, physical role function, and vitality. This pattern of response may signal the differentiation between adaptive, resilient coping responses and less adaptive responses that impact functional status.

Our identification of a link between PTSD symptom clusters and functional status is consistent with a previous report (Pietrzak et al., 2010), though our findings significantly expand upon the prior work both by examining the impact of psychophysiological responses and by looking at a more broadly representative population of SMs, all of whom fell below diagnostic criteria, as opposed to solely focusing on those meeting diagnostic criteria for PTSD (Rauch and Foa, 2006). Our findings complement previous studies that have shown that combat-related VR engenders significant psychophysiological reactions among SMs (Kramer et al., 2013; Costanzo et al., 2014) and decrease with treatment (Rothbaum et al., 2014), as well as that catecholamines play an important role in PTSD and functional status (Young and Breslau, 2004). Similar to more recent work (Freeman et al., 2014), our findings also demonstrate the assessment utility of VR in relation to PTSD. We are able to expand significantly upon the prior literature by exploring relationships between individual catecholamines, PTSD symptom clusters, and various functional domains in a sample of SM with a wide range of symptom severity. We report that norepinephrine modulates the relationship between avoidance and markers of physical functioning (e.g., general physical functioning, physical role functioning, vitality). One previous report of a SM with post-deployment PTSD documented greater epinephrine and norepinephrine responses to combat sounds than was seen in controls (Liberzon et al., 1999), which is not surprising, but our study includes larger numbers and assesses the full spectrum of PTSD symptoms and reveals a far more nuanced picture. Our results support a role for VR in making such assessments, with further studies needed in order to refine its full value.

In the present study, we included SM with a wide range of traumatic symptoms. In doing so, we were able to capture a population of SM who may already be engaging in resilient coping techniques, marked by their capacity to successfully adapt in the face of stress and adversity (Jensen and Fraser, 2005). One subset of our study population of particular interest is a group that had low norepinephrine responses and relatively good functional status despite relatively high avoidance scores. Whereas resilience has more commonly been used to refer to those reporting relatively few symptoms after significant trauma, it may be that we are distinguishing another pattern of resilience in this subset in which they acknowledge symptoms but are not necessarily as troubled by them, either with regard to the autonomic nervous system, or in terms of functional status. Although PTSD had not been labeled as such at the time of World War I or II, it is clear that there are many SMs, as well as such groups as Holocaust survivors, who may have exhibited a similar pattern, with sleep disruptions and other symptoms, yet they were somehow able to function relatively well, and it would certainly have been interesting to see what pattern their catecholamine responses displayed. There are many avenues for future research to investigate regarding resilience in a biopsychosocial framework. Because kernels of resilience are embedded within an individual prior to a trauma (Feder et al., 2009), long-term prospective studies would be especially helpful to further assess the trajectory of catecholamine levels in the face of trauma. Such investigations could help to elucidate why one person develops PTSD and another does not following a traumatic experience.

There are several limitations inherent in our study. First, the cross-sectional nature of the study does not allow us to draw causal inferences. We cannot rule out common method variance that may result with the use of a single questionnaire, and there may also be extraneous variables of significance that were not measured. Therefore, alternative explanations are plausible (e.g., responses to PCL-M items may be influenced by salient events and influence the reportable time-frame). Though our findings require replication in other populations, our results represent a foundational step in utilizing catecholamine response to VR combat scenarios in order to improve our understanding of the relationship between PTSD symptom clusters and functional status.

In recent years, VR has progressed from the realm of science fiction to an accepted and proven method for delivering treatment for PTSD (Goncalves et al., 2012) and advances in inexpensive hardware may soon make it a staple for video gaming. VR has already been utilized to improve our understanding of prospective memory (Gonneaud et al., 2012), attentional processes (Parsons and Rizzo, 2008), attention bias among smokers (Marra et al., 2009), and food cravings (Ledoux et al., 2013), and our work here is in keeping with such intellectual explorations. We demonstrate that the measurement of catecholamine responses to traumatic or stressful virtual presentations can enhance our understanding of PTSD symptomatology, and what really matters most, how well someone is functioning in the various domains of life. Translational approaches to PTSD treatment focused on individual symptom clusters hold promise (Norrholm and Jovanovic, 2010), and we believe that elucidation of the significance of the clusters can be improved by exploring sympathetic nervous system and consequent physiologic responses to VR. Our report represents an early step on the path to improved understanding and more individually tailored approaches to PTSD symptoms.

## Conflict of Interest Statement

The authors declare that the research was conducted in the absence of any commercial or financial relationships that could be construed as a potential conflict of interest.
